# The caregiver’s journey: A qualitative study on the integration of family caregivers of advanced cancer patients in outpatient settings in Germany

**DOI:** 10.1017/S1478951525100242

**Published:** 2025-07-08

**Authors:** Petya Zyumbileva, Ute Goerling, Anne Letsch, Stefan M. Gold, Matthias Rose, Christof von Kalle

**Affiliations:** 1 Clinical Study Center (CSC) of Charité and BIH, Charité – Universitätsmedizin Berlin, Berlin, Germany; 2 Department of Psychosomatic Medicine, Charité – Universitätsmedizin Berlin, Berlin, Germany; 3 Charité Comprehensive Cancer Center, Charité – Universitätsmedizin Berlin, Berlin, Germany; 4 University Cancer Center Schleswig-Holstein, Universitätsklinikum Schleswig-Holstein, Kiel, Germany; 5 Department of Psychiatry and Neuroscience, Charité – Universitätsmedizin Berlin, Berlin, Germany; 6 German Center for Mental Health (DZPG), Berlin/Potsdam, Germany; 7 Clinical Study Center (CSC) of Charité and BIH, Berlin Institute of Health at Charité – Universitätsmedizin Berlin, Berlin, Germany.

**Keywords:** family caregivers, dyad, outpatient cancer care, psychosocial support, Germany

## Abstract

**Objective:**

Family caregivers play a critical yet often overlooked role in healthcare, facing the dual challenge of providing clinical care while managing their emotional well-being. Although several studies have investigated the supportive care needs and services for caregivers of advanced cancer patients integrated into specialized palliative care inpatient units, little is known about cancer caregiver integration and support structures in German outpatient cancer care. This qualitative study addresses this gap by exploring the experiences of family caregivers in Germany, using a dyadic approach to assess their needs, identify referral strategies, and evaluate oncologists’ perspectives on improving caregiver integration and support.

**Methods:**

Thematic analysis was conducted on semi-structured interviews with 14 advanced cancer patients, 15 family caregivers, and 3 oncologists. MAXQDA software facilitated the identification of key themes and codes.

**Results:**

Three interconnected themes emerged: (1) The Impact of Illness on the Dyadic Relationship, (2) Communication with Physicians and Understanding of Healthcare Information, and (3) Challenges and Preferences in Navigating Healthcare Services and Psychosocial Support.

**Significance of results:**

The findings highlight the need for enhanced support in caregiving to improve cancer care quality, emphasizing that early palliative care integration is vital for addressing caregiver needs as a core component of comprehensive cancer care. Healthcare practices should adopt personalized, proactive support strategies from diagnosis, implement regular needs assessments, and leverage digital healthcare tools to enhance the efficacy and efficiency of caregiver support.

## Introduction

Cancer is a major health issue in Germany, with an estimated more than 600,000 new cancer cases in 2022, making it one of the leading causes of morbidity and mortality (Ferlay et al. 2024). One-third of all new cancer cases and deaths are solid tumors, such as breast, colorectal, and lung cancers, which are also among the top causes of cancer-related mortality in Germany (Ferlay et al. 2024). Cancer care involves a complex process that extends beyond the hospital walls and into patients’ homes. Caregiving is crucial to cancer patients and their families (Sun et al. 2019). Family caregivers (FCs), defined here as relatives, friends, or partners who provide unpaid support to cancer patients, play a central role in cancer care (Lambert et al. 2016). FCs facilitate access to treatment, coordinate communication among healthcare providers, help patients understand and cope with medical information, and navigate the complexities of the healthcare system. FCs of advanced cancer patients encounter significant physical, emotional, social, and financial challenges that impact their overall well-being while tackling caregiving tasks, which leads to a dual burden (Goerling et al. 2020; Harrison et al. 2021; Kent et al. 2016; Lambert et al. 2016; Sherman 2019; Stenberg et al. 2010; Sun et al. 2019). Previous studies revealed common unmet needs among FCs in physical, psychological, and informational domains, including a frequent need for psychological support to address anxiety regarding disease recurrence or progression, as well as disease, treatment, and care-related information, making a dyadic approach to advanced cancer care essential (Chen et al. 2016; Crotty et al. 2020; Ferrell and Kravitz 2017; Kim et al. 2020; Ream et al. 2013; Wang et al. 2018; Yang et al. 2021). This approach focuses on the shared assessment and management of illness by patients and caregivers (Lyons and Lee 2018). Advanced cancer patients’ psychological distress has been shown to significantly increase caregivers’ burden, disrupting family dynamics and daily life (Caruso et al. 2017; Johansen et al. 2018; Li et al. 2018; Rhondali et al. 2015).

Despite the importance of routine screening for caregivers’ needs, it is not yet established in Germany (Oechsle 2019; Oechsle et al. 2021). While some studies have focused on FCs in specialized palliative care inpatient units (Ullrich et al. 2021), little is known about caregivers' challenges, needs, and support systems in outpatient cancer settings.

The primary objective of this study is to address this gap by adopting a dyadic approach to explore the experiences and needs of FCs of advanced cancer patients in outpatient settings in Germany. The study also seeks to identify suitable options for routine needs assessments and potential strategies for referring caregivers to appropriate support services, such as psychological counseling, social support networks, and informational resources. By including oncologists’ perspectives, it evaluates professional views on integrating FCs, identifies potential barriers, and explores ways to enhance needs assessments and caregiver support, ultimately improving care quality for both patients and caregivers.

## Methods

### Theoretical background

This qualitative study is grounded in the Theory of Dyadic Illness Management, which views illness management as an interdependent process between the patient and caregiver (Lyons and Lee 2018). This theory aligns with the study’s objectives of promoting regular, dyad-focused needs assessments that consider both the practical and emotional needs of FCs in outpatient cancer care, alongside those of the patient. By including oncologists’ perspectives, the study addresses the contextual factors outlined in the theory, examining available support and systemic barriers to caregiver integration. The framework seeks potential strategies to enhance caregiver support and improve overall dyadic care quality.

### Study design

This study used a qualitative design. The COnsolidated criteria for REporting Qualitative research (COREQ) checklist was utilized for reporting (Tong et al. 2007).

### Participants and recruitment

Participants were selected through collaboration with medical staff at the outpatient clinic at Charité Comprehensive Cancer Centre, using purposive sampling to achieve theoretical saturation (Hennink and Kaiser 2022). Physicians identified eligible patients and caregivers and provided them with detailed study information. Written informed consent was obtained from those interested in participating. Inclusion criteria for the patients comprised: (1) ≥18 years old; (2) being diagnosed with Stage IV solid tumor; (3) undergoing cancer treatment; (4) having a FC; and (5) speaking German. Eligibility criteria for the family caregivers included: (1) being identified by the patient as a primary caregiver providing unpaid care and support; (2) ≥18 years old; and (3) speaking German. The oncologists included in the study were those directly involved in the treatment of the participating patients and were working at the outpatient clinic, purposefully selected to represent clinical experiences relevant to the study’s objectives.

### Data collection

The study included a needs evaluation survey adapted from the NCCN Distress Thermometer (DT) (Mehnert et al. 2006). It covered various areas identified in prior caregiving research, such as the evaluation of emotional, social, financial, and informational needs of FCs, dyadic dynamics, and congruence. Participants, including patients and FCs, completed the survey before their regular consultation with the physician. This was done as a pilot to make their needs visible in the clinical routine. The survey informed the semi-structured interviews post-consultation, aiming to understand participants’ needs, identify gaps and challenges, and discuss additional support modalities vital to caregivers but not initially covered. A detailed description of the study’s tools can be found in the Appendix.

Qualitative interviews were conducted face-to-face in the outpatient clinic between January and September 2023 by the first author (P.Z.), a female PhD candidate in health services and communication research with previous experience and training in qualitative methods. The interviewer had no prior relationship with any of the participants, ensuring a neutral and professional interaction. The first 3 interviews served as pilots, and the interview guide was adjusted according to the experiences. The semi-structured interview guide used in this study is included in the Appendix. Most interviews involved patients and caregivers together, although exceptions were made if caregivers preferred separate sessions or if the patient’s health condition required individual interviews. The dyadic format fostered direct dialog between patients and caregivers, creating an interactive environment reflective of real-life experiences (Froschauer and Lueger 2020). The interviews with the physicians were conducted separately. Basic sociodemographic data (age, gender, and the relationship between caregiver and patient) were collected through brief surveys, while medical information (e.g., diagnosis and treatment status) was obtained from patient records.

### Data analysis

This sociodemographic and medical data were analyzed using basic descriptive statistics in IBM SPSS Statistics 27. The interviews were conducted in German, recorded, transcribed, and translated into English by a bilingual researcher. Reflexive thematic analysis, following Braun and Clarke (2006), was performed using the six stages of data familiarization, inductive coding, initial theme generation, theme refinement, theme definition and naming, and reporting the findings (Braun and Clarke 2006). MAXQDA Analytics Pro 24 was used to organize codes into sub-themes and themes. The first author (P.Z.) conducted an open code of the transcripts, which were then grouped into larger categories to generate central themes. The authors (P.Z., U.G., S.G., C.K.) reviewed and refined these themes to ensure accuracy and depth. Participant confidentiality was maintained using pseudonyms and by anonymizing personal information. The study was approved by the Ethics Committee of Charité University of Medicine Berlin, Germany (EA4/185/22).

## Results

### Population

Fifteen interviews were conducted with 15 dyad couples, including 14 patients and 15 caregivers, with one patient having two primary caregivers (a son and daughter) at the outpatient clinic. While 11 interviews were conducted with the patient and caregiver together, four interviews were held solely with caregivers due to the patients’ health conditions or the caregivers’ requests. A summary of participant characteristics is presented in Table 1. Individual interviews were also conducted with 3 oncologists. The average length of the interviews was 25 minutes (SD = 7, range: 16–44 minutes).

**Table 1.** Patient and caregiver characteristics

	Patient Characteristics* (n = 14) *N* (%)	Caregiver Characteristics (*n* = 15) *N* (%)
Age in years: mean (SD), range	65.9 (±12.3), 39−82	55.5 (±14.9), 28−74
Gender		
Male	7 (50%)	5 (33%)
Female	7 (50%)	10 (67%)
Distress mean (SD), range	5.3 (±2.4), 0−8	4.7 (±2.6), 0−10
Cancer type & stage		
Advanced stage (IV)	14 (100 )	
Colon	5 (36%)	
Lung	3 (21%)	
Gastric	2 (14%)	
Pancreas	2 (14%)	
Hepatobiliary	1 (7%)	
Melanoma	1 (7%)	
Degree of relationship		
Partner		10 (67%)
Adult child		5 (33%)
Living with the patient		
Yes		11 (73%)
No		4 (27%)

* **Note:** Due to rounding, the numbers do not always add up to 100%.

Three main themes emerged in the analysis and are presented together with subthemes and supporting quotes in Table 2 (1a–3z). The results are discussed by theme in the following section.

**Table 2.** Emergent themes and subthemes

Theme	Subtheme	Quotes
**1. The Impact of Illness on the Dyadic Relationship**	1.1 Social Environment’s Influence	***1a*** *“I have four children (…)if things got tough now, they would also support us. So we already have a very good network and it works quite well” (caregiver, daughter, female, 63 years old, Hi005)* ***1b*** *“Yes indeed – our friends are family (…) the groups, for example, the gay and lesbian community, our caregivers, so to speak, are often friends and our circles of friends (…) “(caregiver, partner, female, 69 years old, Hi010)* ***1c*** *“…if someone only has a relationship but no social environment, that can also be a problem. With us now, it’s not that everything ends up in the relationship. We both have people that we talk to – that’s a relief that you don’t have to work everything out in the relationship.” (patient, female, 71 years old, Hi010)*
	1.2 Dyadic Support	***1d*** *“At the beginning, I was at the end of my rope. But for me to accept that and to be able to live and fight with cancer at all, I had to think clearly as well – that’s where my husband helped me, also through the books.” (patient talking about her partner, female, 39 years old, Hi009)* ***1e*** *“I sometimes think a bit negatively. But he is always positive from the beginning, and it’s good that way, because that’s what I need, otherwise it would be even worse for me; it’s already bad enough.” (patient talking about her partner, female, 51 years old, Hi015)* ***1f*** *“What he used to do at home has largely been taken over by me now the entire organization of shopping, household tasks – everything – rests on my shoulders (…) although he is already trying to do some things on his own as far as he can. So, for the most part, the responsibility now falls to me.” (caregiver, partner, female, 62 years old, Hi017)* ***1g*** *“And for me, looking back, I can only say that I would never have survived the time between the discharge date in October and Christmas without my wife and my daughter.” (patient, male, 79 years old, Hi018)*
	1.3 Dyadic Conflict	***1h*** *“The doctor told us …that he won’t get completely well again. It’s a struggle now, and I must tell him that again and again (…) because he says, “I have to get better at some point,” but I say “(…) this is the therapy which you have to endure, where you have to fight” (caregiver, partner, female, 62 years old, Hi017)* ***1i*** *“I did keep something from her once, so ...suspicion of lung metastases (…) And yes, I wanted to go easy on her, in that situation.” (patient about her partner, female, 71 years old, Hi010)* ***1j*** *“Many caregivers complain that the patients themselves, if they always come here alone in between, then they don’t say anything, so communication between caregivers and patients is often not so good.” (physician, A2)* ***1k*** *“Caregiver: but when you described yourself as “lethargic” on the questionnaires (to the patient), that was a relief to me because I had no idea I thought you were depressed, sick, whether you were in pain.. But lethargy, I can understand that.* *Patient: That’s where the questionnaire helped – the question with the feelings, thinking about what feelings I had” (patient, female, 71 years old, and caregiver, female, 69 years old, on the needs assessment tool, partners, Hi010)*
**2. Communication with Physicians and Understanding of Healthcare Information**	2.1 Health Literacy and Information Needs	***2a*** *“Of course, as a person affected, you get something that is told, and sometimes you don’t really notice it, and it would also be important that the caregivers know about it, that you also tell them how it is with the patient” (patient, male, 72 years old, Hi014)* ***2b*** *“That’s why it was important for me that he is always with me because four ears are sometimes better than two” (patient, female, 51 years old, Hi015)* ***2c*** *“And at this moment, the information ‘I have cancer, or my cancer has progressed again’ is so prominent that the information that comes afterward is often not absorbed and processed by the patient very well. And it is very helpful if family, acquaintances or friends are present (…) so that they can categorize it again, to absorb and process this information” (physician, A3)* ***2d*** *“I think that’s actually also important (…)how empathetic the doctor is (…) I would have liked the treating doctor to perhaps show more general therapy options, what might still be possible, what could still be done alternatively” (caregiver, daughter, female, 31 years old, Hi016)* ***2e*** *“We are increasingly having problems with people who have become patients and often lack basic biological understanding. They would need this to understand which therapies we carry out (…). In other words, there is, of course, a need for information. Unfortunately, the need for information is often met by the Internet (...). Much information is not medically proven or proven by medical studies. That is very, very problematic. “(physician A3)* ***2f*** *“I wish to know which symptoms I should or must pay attention to, to be able to assess what is now an alarm signal?” (caregiver, partner, female, 62 years old, Hi017)* ***2g*** *“We just got the doctor’s findings, and we sat down at home and googled, all the time, these terms and abbreviations and stuff. And that was the only thing we had, nothing more. We were able to talk to our general practitioner from time to time because we get on very well with him, he has been looking after us for 20 − 30 years, and he was also able to decipher for us what the doctor’s report said, but unfortunately, we were not taken along at all, and that’s why it would be nice if there were at least some way for caregivers to have these reports explained to them. “(caregiver, daughter, female, 28 years old, Hi018)*
	2.2 Prognostic Communication	***2h*** *“I am a bit different from my husband, I don’t think he wants to know everything – for me, it is the case that I want to know everything, and that helps me more than this carousel of thoughts.” (caregiver, partner, female, 63 years old, Hi006)* ***2i*** *“I often feel that caregivers want to know more about the prognosis than patients themselves. My impression is that many patients don’t really want to know, but relatives are more likely to ask” (physician, A1)* ***2j*** *“What I think is very important is that the oncologists, all oncologists, are psychologically trained. When my husband was diagnosed, he had a few surgeries, etc. (…), then I called the doctor who was treating him at the time, and he said, ‘Why do you want a cure? Your husband only lives half a year,’ and that was on the phone. You don’t need doctors like that.” (caregiver, female, partner, 53 years old, Hi001)* ***2k*** *“The doctors are under tremendous pressure.” (caregiver, partner, female, 67 years old, Hi011)* ***2l*** *“The conversation with the doctor is undervalued in our catalog of services. This also means that doctors don’t have the time to satisfy the patient’s need for conversation adequately. That is a problem.” (physician, A3)*
**3. Challenges and Preferences in Navigating Healthcare Services and Psychosocial Support**	3.1 Caregiver burden and need for psycho-oncological support	***3a*** *“I feel much more burdened now than my husband, and that is quite strange. My husband is doing well, and I’m struggling through it.” (caregiver, partner, female, 63 years old, Hi006)* ***3b*** *”There is also the case that the caregivers appear to be more burdened than the patients themselves.” (physician, A2)* ***3c*** *“The condition in which my daughter and my wife got me out of the hospital in October was not so terrible for me as a person affected; for me, it was a gradual process, but for my daughter and my wife, it must have been a terrible shock what a wreck they got, what they brought home.” (patient, male, 79 years old, Hi018)* ***3d*** *“Yes, it was an absolute shock, and it would have been good, especially at the beginning, if the caregivers had been supported more, because there is a lot of uncertainty (…) And of course, first everything is concentrated on the patient’s well-being, but in the background, the caregivers are simply looking at how they can still survive in this situation, how they can get used to this new reality.” (caregiver, daughter, female, 28 years old, Hi018)* ***3e*** *“I was feeling extremely bad for weeks because he got one complication after the other, and that fed these fears that it could be over in a moment.. I almost feel a little bit traumatized by that experience” (caregiver, partner, female, 63 years old, Hi006)* ***3f*** *“But I have to say that I missed it that someone approached me and asked me about it.” (patient, female, 51 years old, Hi015)* ***3g*** *“…but especially in the beginning I think it would be important to offer and enable more psychological support to the caregivers. And yes, to perhaps reach out to them even more.” (caregiver, daughter, female, 28 years old, Hi018)* ***3h*** *“Yes, I am more worried about her than myself. That my illness is destroying her, this is an area where, for example, psycho-oncological counseling (...) could help a lot. We had not been offered that before” (patient talking about his partner, male, 63 years old, Hi001)* ***3i*** *“Our son is 8 years old, and it hits him very hard that I am sick. And that’s when we looked for help” (patient in a couple-dyad talking about their child, female, 39 years old, Hi009)* ***3j*** *“Psycho-oncology...that would help a lot of patients, but it’s very difficult to get appointments.” (patient, female, 39 years old, Hi009)* ***3k*** *“I think the psycho-oncological services here at the Charité are good. The problem is the crisis intervention in these acute cases. So, when someone is diagnosed as an outpatient and then decompensates psychologically, it’s simply not adequate if the appointment with the psycho-oncologist is three weeks away. “(physician, A1)*
	3.2 Access to Social Services and Financial Support	***3l*** *“For many of those who receive chemotherapy in hospital, there is a kind of social center that gives them advice, for example, to apply for severe disability benefits. Nobody told me about this… I had to find out from friends that you can do that (…). Nobody told me that, and that’s what I missed, I have to say...that there is no place where you can somehow get some information or what possibilities there are to get some financial support. (…) without my partner, I would be standing there now, and it would be difficult financially “(patient, female, 51 years old, Hi015)* ***3m*** *“There are many things that patients don’t even know. You always learn about help, for example, German Cancer Aid, but only through someone, because someone had cancer, and how to apply for a care allowance, and so on.” (patient, female, 39 years old, Hi009)* ***3n*** *“Yes, yes, especially in terms of care, I don’t think that concerns medical treatment, but (I wish) more support.. also with the care degree etc.” (caregiver, daughter, female, 31 years old, Hi016)* ***3o*** *“As far as social services are concerned, we don’t have any access to social services here. That’s certainly the bigger problem. If this is necessary, then we must send the patients to an external center, i.e. to a public facility” (physician, A2)* ***3p*** *“Specifically, in our case,, in the portal outpatient clinic, I have been criticizing for years that no social service works for outpatients. When it comes to social counseling, we have to send patients to the citizens’ offices, which is very time-consuming in Berlin “(physician A1)*
	3.3 Format Preferences and Structure	***3q*** *“One is completely left alone – whether psycho-oncological or alone the question of nutritional counseling (…) The connection doesn’t work (…) Today, after five years, we learned for the first time about nutritional counseling.” (caregiver, partner, female, 53 years old, Hi001)* ***3r*** *“…, but in general, for people who come to the outpatient clinic for the first time, maybe to offer that …as a relative…that you come to the outpatient clinic for the first time or as an inpatient and say “okay, there are possibilities, we can support you here and there, psychosocially and biologically, what do you need? No, I mean, you can always ask the caregivers; maybe they don’t think about certain things, and I would have liked to have had a general overview of where/what if we might need something and where we can find support.” (caregiver, daughter, female, 31 years old, Hi016)* ***3s*** *“Yes, and that’s why I think it would be good, especially at the beginning, for the first steps, for the first weeks, depending on how the disease progresses, whether there are only weeks and months at all.. but especially in the beginning, I think it would be important to offer and enable more psychological support to the caregivers. And yes, to perhaps reach out to them even more.” (caregiver, daughter, female, 28 years old, Hi018)* ***3t*** *“Yes, many questions might have made more sense a little earlier. It’s difficult to offer this help when you’ve already tried to answer most of the questions yourself... only because the questions have come up much earlier.” (patient, male, 72 years old, Hi014)* ***3u*** *“I always join the doctor’s appointment when there is a CT evaluation because that shows the current status; the CT is the decisive criterion to determine the status, how it has developed, positively or negatively. These are the times I am here.” (caregiver, partner, female, 66 years old, Hi004)* ***3v*** *“Once a quarter to half a year, I would say.” (caregiver, partner, male, 36 years old, Hi009)* *„I don’t have a concrete idea – if problems arise, it would be interesting – once a month, or 2 − 3 months is enough now. Unless there are changes, then it would make sense to ask such questions even at short notice.” (caregiver, partner, male, 74 years old, Hi013)* ***3w*** *“(…) we can do it electronically, it’s also faster” (caregiver, partner, male, 36 years old, Hi009)* *“I would do it digitally “(caregiver, daughter, female, 44 years old, Hi014)* *“Well, on the mobile phone or PC yes.” (caregiver, partner, female, 62 years old, Hi017)* ***3x*** *“Yes, such an email address, where you can write about your problem, and one can see if one can call us back and advise us briefly (…) that would be great, such an email box that is simply looked through in the afternoon. I don’t want to barge in during office hours, but it’s also difficult to get a call back again.” (caregiver, daughter, female, 63 years old, Hi005)* ***3y*** *“…email is the best medium for me because I can write when I have time, and patients can reply when they have time (…) With the right technology and if patients are able to handle the technology, it should definitely be done electronically.” (physician, A1)* *“…I think many older people don’t necessarily get on well with digital” (physician, A2)* ***3z*** *“..so to put it briefly, it would be good to have a direct line to the doctor or at least to have a possibility, like in the style of an answering machine, to simply call and say: ‘We would like to know how our father is doing, what the options are right now, what’s next or something.’..so somehow such a consultation or office hours for caregivers just explicitly...and I think that would be quite feasible.” (caregiver, daughter, female, 28 years old, Hi018)*

## Theme 1: The impact of illness on the dyadic relationship

This theme focuses on the complex interactions between cancer patients and their caregivers, considering mutual support and potential conflicts within their relationships.

### Social environment’s influence

The study found that the patient-caregiver relationship was shaped by social factors impacting their well-being. Both groups emphasized the importance of external support networks in managing illness, especially for individuals in the LGBTQ + community, who often relied on chosen families and friends. Such supportive networks were found to be essential for strengthening the dyadic bond by reducing stress and enhancing resilience (1a–c).

### Dyadic support

Dyadic support within patient–caregiver relationships was shown to be important. This included practical support, such as managing household tasks, coordinating medical appointments, handling medication, and understanding health information. Some FCs demonstrated coping strategies involving hope and optimism, which were expressed through emotional encouragement offered to the patient (1d–g).

### Dyadic conflicts

Conflicts were also present in patient-caregiver dyads. Differences in perception of the illness could lead to mismatched expectations. For example, a patient’s optimistic outlook sometimes clashes with a caregiver’s more cautious perspective, causing frustration and emotional tension (1h). Patients sometimes chose to withhold information to shield their caregivers from stress, but this can undermine the trust that is essential for a strong relationship, as noted by caregivers and observed by physicians (1i–j). Addressing the emotional needs of both patients and caregivers was found to resolve conflicts. One patient–caregiver dyad reported that emotional assessments improved their communication and understanding, with the caregiver being reassured when the patient identified as “lethargic” in a survey, after initially assuming depression or pain in the patient (1k). Such examples underscore the value of regular reflexive assessments for both individuals.

## Theme 2: Communication with physicians and understanding of healthcare information

Effective communication among physicians, patients, and caregivers is vital yet challenging. This theme emphasizes caregivers’ role in helping patients process medical information and highlights the importance of health literacy, addressing caregivers’ needs, and adapting prognostic communication strategies.

### Health literacy and information needs

Understanding the disease situation and the treatment is an essential part of physician–patient–caregiver communication. The news of cancer or its progression can be overwhelming. Study participants and physicians highlighted the support given by FCs during interactions with doctors, helping to remember and process medical information (2a–c). Physicians addressed health literacy issues in caregivers and patients – particularly in using the internet as a knowledge source – as a challenge in shared decision-making (2e).

To better support their loved ones, FCs expressed a significant need for improved informational guidance, especially regarding medical reports, as highlighted by a caregiver who voiced the need for more disease-related information to better care for her husband (2f).

### Prognostic communication

The study revealed that patients and their FCs might have different information needs, which presents challenges in discussing prognosis. FCs typically wanted detailed information to prepare for all outcomes, while patients preferred less detail, as perceived by the oncologists (2h–i). FCs tended to adopt a problem-solving approach, actively seeking information and communicating with healthcare professionals to address uncertainties. They mentioned that satisfying interactions with physicians involve empathy and thorough discussions of treatment options (2d, 2j). However, time constraints on doctors were highlighted as a significant barrier to effective communication, as noted by patients and physicians (2k–l).

## Theme 3: Challenges and preferences in navigating healthcare services and psychosocial support

Caregiver burden, as a distinct theme at the individual level, is directly linked to the need for psychosocial support services at a structural level.

FCs face significant challenges when it comes to their needs and accessing healthcare services. While various services are available, there are gaps in the system that must be addressed.

### Caregiver burden and the need for psycho-oncological support

FCs often feel more distressed than the patients, as noticed by both the patients and doctors, frequently prioritizing the patient’s needs over their own well-being (3a–c). Receiving a cancer diagnosis is especially shocking for most FCs, leading to feelings of helplessness, anxiety about the illness's progression, uncertainty regarding the prognosis, and concerns about changes in their own lives (3d–e). They expressed a need for more proactive psychological and social support to be offered earlier in the treatment process (3f–g).

Service integration issues were noted as some patients and caregivers were unaware of available psycho-oncological services, especially in outpatient care (3h). Younger couples with small children face unique challenges when one parent is diagnosed with cancer, as they must balance childcare responsibilities with caregiving (3i). Discussing the illness with their children can also be difficult, and long waiting times for psychological support further complicate these issues, leaving families without timely assistance during critical moments (3j). Oncologists identified this as a structural problem, recognizing the need for early assessments and immediate support following a diagnosis (3k).

### Access to social services and financial support

The study revealed a clear need for better access to information regarding social benefits, financial aid, and other social services (3l–n). Participants emphasized the importance of proactive outreach and comprehensive lists of available resources. Physicians recognized the lack of social services in outpatient settings as a significant structural issue (3o–p).

### Format preferences and structure

FCs stressed the need for better communication and support in the care process and emphasized challenges in accessing help due to structural barriers, indicating the need to address gaps in the system (3q–r). Early needs assessments were preferred by FCs, along with the implementation of a referral system for supportive services (3s–t). They suggested regular evaluations every 3–6 months or during significant treatment milestones, such as staging procedures or in cases of problem occurrences (3u–v). Most participants appreciated email communication for its flexibility, and FCs favored proactive support and digital tools (3w–x). While physicians found digital communication effective, they noted that paper-based options might still be necessary for older patients (3y). Improvement suggestions included establishing direct communication lines, voicemail options, and designated office hours for caregiver consultations (3z).

## Discussion

This study explores the experiences and needs of FCs in advanced cancer care within German outpatient settings, identifying key themes: (1) The Impact of Illness on the Dyadic Relationship; (2) Communication with Physicians and Understanding of Healthcare Information; (3) Challenges and Preferences in Navigating Healthcare Services and Psychosocial Support. A unique aspect of the study is its inclusion of doctors’ preferences and the obstacles faced in providing psychosocial support, aiming to improve communication and collaboration among patients, caregivers, and oncologists. The research highlights the necessity for tailored support strategies and better resource awareness for FCs in this context.

While previous studies primarily focused on caregivers, this project integrates both patient and caregiver perspectives into a unified needs assessment framework. This approach highlights dyadic incongruence, such as differing views on the disease, emphasizing the interdependent nature of their needs. Unlike one-sided assessments, this method offers a more comprehensive view of support needs in palliative care. Findings suggest that regular joint assessments can address mismatched expectations and unmet needs, enhancing mutual understanding and care quality. Advance care planning is essential for aligning patient and caregiver expectations and reducing uncertainty about future needs. Early systematic integration of palliative care and structured conversations should be integrated into cancer care trajectories and can enhance care quality, lessen uncertainty, reduce caregiver burden, and ensure alignment with the patient’s values and preferences (El-Jawahri et al. 2024; Hoerger and Cullen 2017; Pini et al. 2022). While a recent German study examined doctors’ perceptions of involving caregivers in elderly care decision-making (Heidenreich et al. 2023), little is known about oncologists’ preferences for evaluating and addressing FC’ needs in routine care. A distinctive aspect of this study is its inclusion of physicians’ preferences and barriers in implementing psychosocial support structures. Given the specific outpatient setting, the study suggests effective caregiver integration strategies that accommodate the preferences of all three parties, ultimately providing tailored recommendations for enhancing the caregiving experience.

The current findings align with the existing literature, adding evidence to the previous studies. The project highlights significant emotional and informational challenges faced by FCs, corroborating prior research on their high caregiving burden and unmet needs (Bevans and Sternberg 2012; Girgis et al. 2013; Semere et al. 2020). It underscores the critical role of the social environment in providing practical and emotional support (Jiang et al. 2022). The social environment influences not only the well-being of patients and caregivers individually but also shapes the dynamics of their dyadic relationship. Positive social support can enhance dyadic coping by promoting shared understanding and reducing conflict, while a lack of support can negatively contribute to tension within the dyad.

The study emphasizes the significance of healthcare providers promoting transparent communication between patients and caregivers to improve their mutual understanding and disease management. It recommends tailoring communication to meet the specific needs of patients and caregivers, including effectively managing the complicated dynamics of sharing prognosis information. The study confirms prior research indicating misunderstandings about prognosis among cancer patients and their caregivers and highlights the importance of considering discordant prognostic information preferences within the patient-caregiver relationship in advanced cancer care (Applebaum et al. 2018; Gray et al. 2021; van der Velden et al. 2023). The findings support the previous conclusions that caregivers and patients can have different prognostic awareness (Applebaum et al. 2023; Diamond et al. 2017).

Furthermore, empathetic communication and support for disease understanding and prognostic awareness were vital aspects of the interaction between physicians, patients, and caregivers. This finding confirms the results of a previous study that highlighted the importance of such support throughout the cancer trajectory (Preisler et al. 2018). Additionally, the current study confirms earlier findings on substantial unmet information needs of caregivers concerning treatment, side effects, and symptom management (Cochrane et al. 2021). Beyond discussing treatment options and results, however, it is crucial for physicians to address end-of-life issues, explore patients’ values and preferences, and integrate palliative care early in the cancer care continuum to support quality of life. This holistic communication is essential to ensure that care aligns with the goals and desires of both patients and FCs.

The study introduces a dyadic approach to assess the needs of patients and caregivers within a shared framework, either together or separately, based on contextual considerations, and incorporates these elements into the established distress evaluation framework (Mehnert et al. 2006). Linking the Theory of Dyadic Illness Management to the study’s results clarifies the shared coping strategies of cancer patients and caregivers, emphasizing the role of social networks and practical support in building resilience. The findings highlight “dyadic support,” where caregivers assist with medical appointments and household tasks, underscoring the importance of practical assistance. Emotional coping, such as caregivers fostering hope, shows how shared positivity helps through challenges. However, optimism can have a dual role – both supportive and possibly hindering shared decision-making through overestimation of benefits – which was not explicitly addressed in the interviews and warrants further exploration in future research. Conflicts from differing illness perceptions underscore the importance of enhancing communication and anticipation. This can be accomplished through advance care planning programs, which foster understanding between patients and caregivers.

Integrating caregivers’ assessment needs into routine care in German cancer outpatient settings aims to increase their visibility. While patients and caregivers welcomed needs assessments, timing is crucial, with a preference for early needs evaluation and immediate psychological support after diagnosis. The study revealed challenges regarding the integration of psychological services. Structural barriers, such as long waiting times and insufficient social services integration, must be addressed to improve care quality in outpatient settings. FCs preferred personalized and accessible support, including digital formats, indicating a demand for innovative solutions. These results are consistent with prior research highlighting the potential of new technologies in supporting caregivers across various disease contexts, such as dementia caregiving (Gris et al. 2023). While innovative tools provide valuable support, they must be viewed as supplements to, not replacements for, human-centered approaches that address caregivers’ needs. This study’s strengths lie in its comprehensive coverage of a wide range of experiences across different types of advanced solid tumors and age groups, including younger cancer patients and FCs who might face different challenges than older groups (Justin et al. 2021). Additionally, incorporating the perspectives of physicians enriches the study. Qualitative methodology provides insights into participants’ experiences, and including LGBTQ + members added diversity to the study. This group often faces unique challenges, such as increased life stress and limited family support (Power et al. 2022). The experiences of the LGBTQ + dyad in our study suggest a potential need for further research to explore the specific needs and resources of this population in more detail.

### Limitations and future directions

This project has several limitations. Conducted at a single center with a qualitative approach, its findings have limited transferability. Most interviews included both patients and FCs, but some included only FCs, which may affect the results. Dyadic interviews might have restrained FCs from sharing sensitive emotions, and the limited sociodemographic data hinders our understanding of diverse caregiving contexts. Future research should include a broader range of sociodemographic variables. The study focused on German-speaking FCs of stage IV cancer patients, excluding earlier stages and non-German speakers, which limits transferability. It also concentrated on FCs of advanced cancer patients undergoing active treatment, who may perceive prognosis differently than those caring for patients receiving only palliative care. This distinction is crucial for interpretation. Future research should involve FCs from all disease stages for a broader range of supportive and palliative care experiences and consider diverse linguistic and cultural backgrounds. Many patients and FCs reported their previous experiences retrospectively, potentially introducing bias. This highlights the need for future research to capture more immediate and unbiased insights into their initial needs.

This study focuses on the German healthcare system but may also have implications for outpatient care in other countries, as FCs of cancer patients often face similar challenges. However, cultural and systemic differences are important to consider. Future research should examine how caregiver needs are addressed in various cultural and healthcare settings to develop effective, context-sensitive interventions. Cross-cultural studies can aid in developing targeted interventions and policies, especially in resource-limited regions.

## Conclusion

The findings of this study have significant practical implications for healthcare practice and policy. Healthcare systems should adopt a caregiver-inclusive approach that guarantees early inclusion of palliative care, provides easy access to information, proactive psychosocial support from the start of the cancer journey, and tailored communication with healthcare providers.

## Supporting information

10.1017/S1478951525100242.sm001 Zyumbileva et al. supplementary material Zyumbileva et al. supplementary material

## Supplementary material

The supplementary material for this article can be found at https://doi.org/10.1017/S1478951525100242.
