# Supplementary material for: The caregiver’s journey: A qualitative study on the integration of family caregivers of advanced cancer patients in outpatient settings in Germany
Source: Palliat Support Care. 2025 Jul 8;23:e128. doi: 10.1017/S1478951525100242 (PMC13166349; doi:10.1017/S1478951525100242)
Supplement: Zyumbileva et al. supplementary material [file S1478951525100242sup001.docx]

Appendix

# NCCN Distress Thermometer (DN) and Survey

## Survey (Distress and Needs Assessment) *– translated from German*

### Instructions for Family Caregivers

A. Please circle the number on the thermometer to the right (0-10) that best describes how burdened you felt over the past week, including today.


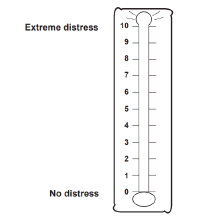


B. Please indicate whether you have experienced problems in any of the following areas* over the past week, including today. For each area, check YES or NO or fill in the fields accordingly.

### General Information

Age

Gender

 Male  Female  Other  No information provided

Relationship to the patient:
(e.g., partner, daughter, son, parent, etc.)

### Areas*

#### Emotional Aspects

What is currently the greatest challenge in your daily life?

______________________
______________________

Name the three most common emotions that you have experienced recently (last week)

______________________
______________________

#### Informational/Health-Related Aspects

Do you have any unanswered questions or difficulties regarding:

Coordination of medical care ☐ YES ☐ NO

Transportation / Mobility ☐ YES ☐ NO

Wound care / Lymph drainage / Stoma etc. ☐ YES ☐ NO

Nutrition ☐ YES ☐ NO

Medication therapy ☐ YES ☐ NO

Other (or briefly describe your question or problem)

______________________
______________________

How would you rate the patient’s current health situation? (Scale 1 (very poor) – 5 (very good))

______________________

#### Social/Dyadic Aspects

Have there been any issues or conflicts between you and the patient recently?

If yes, to what extent?

______________________

From your perspective: what does the patient currently need the most?

______________________

#### Practical/Financial Aspects

Housing ☐ YES ☐ NO

Employment / School / Training ☐ YES ☐ NO

Childcare ☐ YES ☐ NO

Has your professional/financial situation changed? ☐ YES ☐ NO

If yes, to what extent? ______________________

### Instructions for Patients

A. Please circle the number on the thermometer to the right (0-10) that best describes how burdened you felt over the past week, including today.


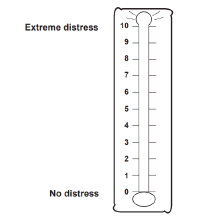


B. Please indicate whether you have experienced problems in any of the following areas* over the past week, including today. For each area, check YES or NO or fill in the fields accordingly.

### General Information

Age

Gender

 Male  Female  Other  No information provided

### Areas*

#### Emotional Aspects

What is currently the greatest challenge in your daily life?

______________________
______________________

Name the three most common emotions that you have experienced recently (last week)

______________________
______________________

#### Social/Dyadic Aspects

Have there been any issues or conflicts between you and your relatives recently?

If yes, to what extent?

______________________

From your perspective: what do you currently need the most?

______________________

#### Practical/Financial Aspects

Housing ☐ YES ☐ NO

Employment / School / Training ☐ YES ☐ NO

Childcare ☐ YES ☐ NO

Has your professional/financial situation changed? ☐ YES ☐ NO

If yes, to what extent?

______________________

#### Physical Problems / Health-Related Aspects

Pain ☐ YES ☐ NO

Nausea ☐ YES ☐ NO

Fatigue ☐ YES ☐ NO

Sleep ☐ YES ☐ NO

Movement / Mobility ☐ YES ☐ NO

Washing / Dressing ☐ YES ☐ NO

Physical appearance ☐ YES ☐ NO

Breathing ☐ YES ☐ NO

Mouth inflammation ☐ YES ☐ NO

Eating/Nutrition ☐ YES ☐ NO

Digestive problems ☐ YES ☐ NO

Constipation ☐ YES ☐ NO

Diarrhea ☐ YES ☐ NO

Changes in urination ☐ YES ☐ NO

Fever ☐ YES ☐ NO

Dry / itchy skin ☐ YES ☐ NO

Dry / stuffy nose ☐ YES ☐ NO

Tingling in hands/feet ☐ YES ☐ NO

Feeling swollen / bloated ☐ YES ☐ NO

Sexual problems ☐ YES ☐ NO

Do you have any open questions regarding your treatment?

______________________

Other (or briefly describe your question or problem)

______________________

How would you rate your current health situation? (Scale 1 (very poor) – 5 (very good))

______________________

# Guidelines for the Dyadic Interviews

## Topic List

Note: These interview guidelines serve as a framework for an in-depth exploration of participants' experiences and perspectives while allowing flexibility for interviewers to tailor the conversation to the specific context.

- Experience with Completing the Survey and Preferences for Needs Assessment Formats

Reflections on any challenges or difficulties encountered during survey completion, including ease of understanding, preferences for the survey format, frequency, and delivery for a regular need assessment, and suggestions for future improvements to enhance accessibility and effectiveness. *(e.g. How did you experience filling out the survey? Were there any specific difficulties you encountered with the format or content – if yes, which? What suggestions would you have for improving the survey format, delivery method, or frequency of completion? When, in your opinion, is the best timing to evaluate the needs of family caregivers, and how often should this be done?)*

- Coverage of Important Topics and Identification of Unmet Needs and Challenges

Assessment of whether the survey adequately addresses essential topics relevant to the needs of family caregivers, along with identification of critical areas or topics missing from the survey or routine clinical discussions and additional areas participants feel should be included in future assessments. *(e.g.* Did the surveys cover all aspects and topics that are important to you as a caregiver? If not, which topics do you feel were missing? What additional areas would you like to see included in future needs assessments? Which topics are particularly crucial to you (as a patient-caregiver dyad / explicitly as a caregiver)?

- Experiences with Medical Care and Communication with Physicians

Experience and interactions with healthcare providers, clarity of communication, and attention to key concerns (*e.g. how do you experience the communication with the physician from a caregivers’ / patients’ perspective? And concretely about your specific needs?)*

- Preferences for Support Structures

Discussion of potential improvements to better support family caregivers and patients as a dyad, including recommendations for changes to healthcare system practices, along with the need for effective communication and practical support systems that could benefit both caregivers and patients within routine care settings. (*e.g. Do you currently feel in need of any additional support? What forms of support would you wish were available to you? What specific recommendations do you have for improving the situation for the patient and caregiver as a dyad within the healthcare system?)*

# Guidelines for the Interviews with the Physicians Topic List

**Note:** These guidelines serve as a flexible structure for exploring physicians' perspectives on caregiver involvement and psychosocial support, adaptable to the unique context of each interview. They allow physicians to share their insights in an open and reflective manner.

Focus on the following topics:

- Perception of Family Caregivers’ Role in Treatment

Reflections on family caregivers’ role in the treatment process, including their impact on patient care, contributions, and challenges they may present in care delivery *(e.g. How do you perceive the role of family caregivers in the treatment process?)*

- Differences in Information Needs of Patients and Family Caregivers

Observed differences in information needs between patients and caregivers, including patterns or specific areas where caregivers may need additional or different information than patients. (e.g. *How do you assess the information needs of the patients and their caregivers – are there differences? Have you observed differences in the desires for prognostic information between patients and their caregivers? If so, what are they?)*

- Perception of Emotional Challenges Faced by Family Caregivers

Perception of emotional reactions and challenges commonly experienced by caregivers, especially around the time of diagnosis; strategies for addressing caregivers’ emotional needs, along with any challenges or limitations encountered when engaging with caregivers’ emotional responses over time*. (e.g. How do you perceive the emotional experiences and reactions of relatives upon learning of the cancer diagnosis? What challenges do you personally see in dealing with caregivers?)*

- Effective Communication and Caregiver Involvement Strategies

Discussing techniques and preferences for communication ways with patients and family caregivers, including strategies and challenges for involving caregivers in treatment discussions as well as unmet needs observed among family caregivers, and current approaches for supporting caregivers within the clinical setting. *(e.g. which communication methods do you find best when interacting with cancer patients and their family caregivers? Are there specific strategies that you use when discussing treatment plans with patients and their relatives, and if so, what are they? How do you currently address or support caregivers in the treatment process? What would you like to improve to involve better or support caregivers?)*

- Integration of Psycho-Oncology and Psychosocial Support

Importance of integrating psychosocial support and psycho-oncology services into routine care, including optimal timing and frequency for such assessments; discussion of potential barriers to integrating psychosocial services, along with recommendations for addressing these challenges to better support both caregivers and patients. *(e.g. how do you envision the integration of psychosocial assessment and support into the treatment process? What barriers or challenges do you see in effectively integrating psycho-oncology and social services in outpatient care?)*
